# Supplementary material for: Patient‐specific daily pretreatment setup protocol using electronic portal imaging for radiation therapy
Source: J Appl Clin Med Phys. 2005 Nov 22;6(4):1–13. doi: 10.1120/jacmp.v6i4.1954 (PMC5723453; doi:10.1120/jacmp.v6i4.1954)
Supplement: Supplementary file 1 — Supplementary Material Files [file ACM2-6-001a-s001.doc]

Patient-specific daily pre-treatment setup protocol using electronic portal imaging for radiation therapy

Running Title: EPID-Based RT Setup Correction

Patient-specific daily pre-treatment setup protocol using electronic portal imaging for radiation therapy

**Abstract:**

Purpose: Evaluate electronic portal imaging (EPI) as a means to identify and correct field displacement in patients with problematic external beam radiotherapy setups.

Materials and Methods: 14 patients with problematic setups were identified for pretreatment daily EPI beam monitoring as part of a physician-directed therapist intervention protocol. Pretreatment EPIs were used to re-align fields as necessary to bring the setup within the physician-prescribed tolerance level. For comparison, daily EPIs were available for 12 “control” patients who had no particular setup difficulties and for whom on-line beam re-alignment was not made. Anatomy matching software was used to measure setup variation along medial-lateral, superior-inferior, and anterior-posterior axes.

Results: On-line field re-alignment yielded a significant (p = 0.001) improvement when comparing initial and final setup variations. The mean standard deviation of setup displacement averaged over three axes was reduced from 6.4 mm to 3.1 mm after realignment. The final variation of protocol patients was comparable to that of control patients.

Conclusions**:** EPI provided effective means to perform on-line beam re-alignment in a group of difficult to position patients. This procedure resulted in a reduction in setup displacement that was statistically significant, clinically relevant, and approached that of a more typical patient group.

**Key Words**: Electronic portal imaging, setup error, radiotherapy, quality control

**PACS numbers**: 87.53.Oq (Portal Imaging in Therapy)

#### Introduction:

The goal of radiotherapeutic treatment for cancer is to reliably provide optimal target coverage and dose level to the tumor while minimizing the toxicity to critical normal organs. Improved radiotherapy planning and treatment methods such as three-dimensional (3D) conformal or intensity modulated radiation therapy will come closer to achieving this goal if the actual dose distribution approaches that of the treatment plan. However, actual dose distribution may deviate from the treatment plan as a result of systematic and random errors in patient postioning, as well as internal target displacements. Increasing the accuracy of radiation dose delivery to the intended target should improve the tumor control probability and reduce treatment-related morbidity (1-8).

Pretreatment patient positioning constitutes one important element in determining treatment accuracy. Currently, weekly port films are a standard method for assessment of patient positioning accuracy (9). Numerous studies, however, reported significant errors in patient setup and treatment delivery (10-17), that may not be recognized with the standard approach. Furthermore, it has been suggested that more frequent port imaging could improve setup accuracy, and thus improve clinical outcomes (18, 19).

The electronic portal imaging device (EPID) provides a possible means by which patient setup accuracy could be more vigilantly monitored (20, 21). By use of an EPID, on-line digital port images may be efficiently captured and analyzed before, or even during, every treatment session. For patients who have no particular setup difficulties, this high level of monitoring may be unnecessary. However, for a subset of radiotherapy patients that tend to have large random setup variations, daily EPID on-line monitoring with on-line correction may result in significant improvements in treatment accuracy (22) and an improved risk-benefit ratio. The purpose of the present study was to evaluate the utility of the EPID to identify and correct pretreatment patient setup deviations from the intended target volume in patients with known setup difficulties. To serve as a comparison measurement of daily setup variation, daily EPIs were taken for a separate random group of patients having no particular setup difficulty. While this group was imaged daily, no attempt to correct positional variation was made before or during that day’s treatment. For this control group, setup accuracy was verified by the standard practice of physician review of weekly portal images.

Past studies that have utilized EPID-based on-line setup corrections to reduce random setup uncertainty have tended to use relatively complex treatment protocols including full 3D matching, combined modality imaging and sophisticated algorithms for analysis. These protocols have proven to be time-consuming and difficult to implement in practice (23-26). The protocol in the present study was designed to provide a simple and unobtrusive method of improving setup accuracy in difficult to position patients. After the physician designed a patient-specific prescription for tolerances of setup variation, therapists carried out the protocol, with minimal physician involvement in the day-to-day setup monitoring and correction. These results are compared to a control group who had no particular problems with positioning. This study demonstrates that a simple, easily implemented therapist protocol can significantly reduce setup variation in difficult to position patients.

## Methods:

### Protocol Patients

Between September 1999 and January 2001, 14 consecutive patients treated with external beam radiotherapy (RT) were enrolled into a physician-directed therapist intervention protocol because portal films of the field(s) repeatedly demonstrated clinically significant displacement of the RT field position that results in unacceptable treatment reproducibility. The RT fields were designed by conventional or computed tomography (CT) based simulation and the localization coordinates were transferred to the linear accelerator. Patient positioning was performed by 3-point laser alignment of external (skin) reference points in standard fashion. Field verification by portal film image or EPI was begun with the first treatment and was used to compare the position of bony anatomical landmarks with their location on the simulation radiograph(s). Review of the initial portal images disclosed clinically significant random RT beam displacements and uncertainties in field alignment. This resulted in a situation where frequent portal imaging and field re-alignment was required to reduce inaccuracies in field positioning. As a result, the daily pretreatment EPI protocol was introduced in an attempt to improve the accuracy and consistency of field positioning.

After identification of difficult to position patients, demographic and treatment-related data was obtained to characterize this study population. Eleven patients were female, 3 were male, and all but 1 was treated with curative intent. The median age was ­­73 years (mean, 71; range, 58 -78). 2 - 4 fields (median, 4) were treated with 6 MV or 18 MV photons using multi-leaf collimation (12 patients) or Cerrobend (2 patients) to treat each field each day. The most common field arrangement was 4 field (9 patients), whereas 2 patients were treated by opposed lateral fields and 3 patients by other field arrangements (2 –4 fields) to achieve a median dose of 50.4 Gy (range, 28.8 – 70.2) in 28 fractions (range, 5 - 39). Further characteristics of the study cohort are shown in Table 1.

### Treatment Protocol

Once entered on protocol, each patient had individualized tolerances for setup variation prescribed by the radiation oncologist. Factors influencing the magnitude of the physician prescribed tolerance for setup variation include field size, treatment site, target volume setup margin, and perceived difficulty of patient setup*.* The tolerance for setup correction varied from 5mm to 10mm. Before each treatment, patients were immobilized using a site specific device per clinic routine and then aligned using the external reference points and the coordinate system of the therapy unit. An anterior-poster (AP) or lateral 6 MV photon EPI of the treatment field was then obtained. Field displacement was measured by visual comparison of bony landmarks and field edges in the EPI relative to the simulation radiograph with Varian PortalVision anatomy matching software tools on the EPID, or by visual comparison of bony landmarks and field edges in the EPI relative to the simulation radiograph. If the pretreatment EPI revealed alignment inaccuracies in excess of physician prescribed tolerances, the patient was repositioned and one or more additional EPIs were obtained to assure the field was aligned within the pre-determined specifications. Five-hundred sixty-seven EPIs were obtained during 239 treatment fractions and displacements measured along two of the three mutually perpendicular axes were reported for each. Reported here are setup variations from the first EPI, representing initial setup variation, and setup variations from the final pretreatment EPI before treatment was administered, which is assumed to be the location of the field at treatment time.

EPIs were acquired with either a liquid ion chamber EPID (Varian PortalVision SLIC Mark II, Palo Alto, CA) or amorphous silicon EPID (Varian PortalVision aS500, Palo Alto, CA). The surface of the detector element on the imager was positioned at 140 cm from the source. Field-only, single exposure EPIs were taken if adequate anatomical visualization was present to facilitate analysis. If anatomic visualization was inadequate, a double exposure EPI was obtained.

### Control Patients

For comparison, EPIs were also obtained for 12 “control” patients treated between January 1999 and January 2001 who did not present particular setup difficulties. These patients were treated with curative intent for localized (American Joint Committee on Cancer Stages I-III) carcinoma of the prostate (10 patients) or rectum (2 patients). AP, posterior-anterior (PA), and right and left lateral field-only images were acquired daily, but these images were not inspected on-line and on-line beam realignment was not performed. Nine-hundred fifty-three images from 309 treatment fractions were recorded and analyzed for this group.

*Method of Analysis*

After completion of RT, the simulation radiographs and the EPIs from both groups of patients were transferred to a computer workstation for off-line analysis in the present investigation. For difficult to position patients, the EPID’s anatomy matching software (Varian PortalVision 6.0) was used to measure setup variation along the medial-lateral (ML) and superior-inferior (SI) axes for AP views, and along the AP and SI axes for lateral views. The two step process included an automated initial match, followed by a visual and final edit of the match by the therapists. In addition to setup variation, the number of EPIs taken was also recorded.

Statistical calculations were carried out using Microsoft Excel 97 SR-2, which were confirmed using the JMP 4.0.4 statistical package. The mean setup variation and the standard deviation (SD) of the setup variation were determined in each direction for the initial and final EPIs for each fraction. The mean was assumed to represent systematic variation, while the SD was assumed to represent random variation. Statistically significant differences between the mean variations and SDs were investigated using a two-tailed, paired Student’s *t*-test. Furthermore, the possibility of a correlation between ML, SI, and AP setup variations in the same patient on the same day was also investigated by plotting ML vs. SI displacement, SI vs. AP displacement, and AP vs. ML displacements. No such correlation was found to exist, i.e., the data points were found to vary independently (data not shown).

*Reproducibility*

To test the reproducibility of the field displacement measurements, four protocol patients were selected (patients 2, 5, 6, and 12 from Table 2) for whom the same observer performed the measurement process. Patients 2, 6, and 12 were selected because they were subjectively considered to be of average EPI-to-radiograph matching difficulty as primarily judged by field size and the amount of anatomy visible. Patient 5 was subjectively considered to be the most difficult of the protocol patients to match. The results from the first setup measurement were not used in selecting these four patients.

**Results:**

##### Protocol Patients

An average of 2.4 EPIs per treatment field (range, 1-7) was recorded in problematic setup patients, with EPIs obtained on an average of 14.9 days (range, 5 - 25). On average, beam realignment was performed on 7.7 (52%) of those days. Figure 1 shows the frequency that repositioning was required by the protocol for the difficult to position patient group. A median of 134 MU (mean, 155.7; range 28-380) in addition to the therapeutic dose (Median MU 4820) was administered for the purpose of EPI in this protocol.

Table 2 shows the initial and final SD and maximum displacements in all relevant dimensions for individual patients, as well as the percentage of days each patient was re-imaged and repositioned. The difference between the initial and final setup variations in problematic setup patients was significant (p=0.001). Indeed, the average SD of variation along the three axes in problematic setup patients was reduced from 6.4 mm to 3.1 mm (p=0.001) after realignment, for a 52% reduction in the magnitude of the setup displacement. Over all patients, the SD of displacement for ML was reduced from 6.9 to 3.0 mm (p = 0.001), from 5.9 to 2.8 mm (p = 0.15) for SI, and from 6.5 to 3.9 mm (p = 0.10) for AP directions. Figures 2 and 3 illustrate graphically the reduction in positioning variation achieved.

Similarly, the change in maximum field displacement for ML (28.1 to 15.2 mm), was also significantly (p = 0.001) reduced. Maximum field displacement changes for SI (100.8 to 17.0 mm) and AP (22.4 to 12.2 mm) were reduced markedly on the days with the greatest setup error, and showed a trend toward reduction overall (p = 0.17 and p = 0.13, respectively). Figures 4 and 5 show the extent to which maximum displacement was reduced for each patient after the protocol was put in place.

The overall mean setup variation (a measure of systematic variation) was also calculated, and was found to be very small: 0.4 mm initially, and 0.2 mm after use of the protocol.

The results presented in Table 2 illustrate a substantial difference in the benefit individual patients received from the EPI interventional procedure. In Table 3, the problematic setup group is divided into those who required beam re-alignment during more than 50% of treatment days, and those who had re-alignment less frequently or not at all. For frequently re-positioned patients, the SD of the setup variation, averaged over all 3 directions, was reduced from 8.53 to 3.52 mm (p<0.02). For those re-positioned in less than 50% of treatments, a more modest but still significant reduction from 4.56 to 2.73 mm (p<0.02) in the SD was observed.

### Control Patients

For control patients, 1 portal image was obtained for every field every day, except in cases of technological failure. As shown in Table 3, the SD of the setup variation in the ML direction was 1.8 mm (maximum 7.5 mm), 1.7 mm (maximum 10.8 mm) in the SI direction and 2.7 mm (maximum 11.9 mm) in the AP direction. Overall, the average SD of the setup variation in all directions for this group was 2.1 mm.

*Reproducibility*

For four of the patients identified as difficult to set up, the matching process was repeated by the same observer at a later time, and the two sets of data were compared. The average of the absolute difference between the first and second measurement trial was 0.9 mm. The results are shown in Table 4.

###### Discussion:

Significant setup displacements in radiotherapy patients have been observed in numerous studies (8, 11-15, 26-30). Results of these studies have shown that up to 50% of initial fields are in error and in need of correction. The existence of these setup displacements require that radiation oncologists add a margin around the desired target in order to be certain that the entire target receives the prescribed dose. The addition of these margins, however, increases the likelihood that normal tissue will be affected by high radiation doses, thus increasing the morbidity rate associated with the treatment (see for example ref. 1).

The use of an on-line EPID to identify and correct setup displacements before administration of the full RT dose has been investigated as a possible means to increase tumor control and reduce treatment related morbidity (10, 22, 25, 26, 31-35). These studies have shown significant improvements in the accuracy of patient positioning. However, daily on-line EPID imaging is not utilized in many treatment centers, primarily due to the perceived complexity and time-intensive nature of their use.

In the present study, patients with setup difficulties were identified for frequent EPID-based, on-line monitoring of beam alignment. These patients were placed on a physician-prescribed therapist-intervention protocol that was carried out before each treatment session. The protocol was designed to be as simple and easy-to-implement as possible. For the 14 problematic setup patients in this study, on-line EPID monitoring demonstrated clinically significant setup displacements in which beam re-alignment occurred on over 50% of treatment days. This finding is higher than that observed on most previous studies, but is explained by the fact that the protocol patients were selected on the basis of difficulties encountered early in the treatment course.

In the group of problematic setup patients, it is noteworthy that the mean SD of the initial setup displacement (i.e., random error) was reduced by over 50% after EPI identification and field re-alignment. The reduction in the mean SD along all three axes from 6.4 mm to 3.1 mm was highly significant (p=0.001). Comparing these results with other studies, we find only one other study where patients were selected for EPID monitoring on the basis of perceived setup difficulty (22). In this report of one obese patient treated with pelvic irradiation, the SD of the setup displacement was reduced from 7.9 mm at initial setup to 4.7 mm after correction. Other studies that did not select patients on the basis of setup difficulty showed smaller but still remarkable reductions in random variation. For 14 pelvic irradiation patients with EPIs of AP fields only, Stroom et. al. (35) reported a reduction in mean SD from 2.3 mm to 1.6 mm in the lateral direction, and from 3.0 mm to 1.8 mm in the SI direction. For 16 patients receiving thoracic irradiation and EPIs for AP fields only, Van de Steene et. al. (25) reported a comparable reduction in mean SD from 2.7 mm to 1.5 mm in the lateral direction, and from 3.5 mm to 1.2 mm in the SI direction. While the selection threshold and action level differs somewhat for all these studies, a pattern emerges that demonstrates the feasibility of selecting patients who will gain substantial benefit from EPID-based treatment monitoring and field correction.

The present study also differs from prior studies in that we also evaluated field malalignments in a (control) group of patients without field reproducibility problems. When the setup displacement after on-line correction in protocol patients is compared with the setup displacement of the control group, it can be seen that the displacement in protocol patients approached that of the control patients – that is, we achieved a degree of field alignment accuracy that is acceptable by current standards. The final mean SD of the setup displacement in all directions for protocol patients was reduced to 3.1 mm (Table 2), as compared to a mean SD of 2.1 mm in the control group. While this difference remained statistically significant (p=0.001), the reproducibility studies described above indicate that a 1 mm difference may be too small to be clinically meaningful.

The reduction in setup displacement was even greater in the subgroup of protocol patients who required more frequent beam realignment (>50% of treatments). For this subgroup, the mean SD of setup displacement was reduced from 8.5 mm to 3.5 mm (p<0.02). In particular, on-line EPID monitoring proved especially useful in detecting and correcting occasional large setup displacements, some as great as 10 - 100 mm. Although therapeutic outcome-based studies would be necessary, improvements in setup accuracy of this nature would be apt to beneficially affect clinical outcomes. In addition, further research into identification of subgroups of patients who are most likely to benefit from EPID on-line monitoring may allow earlier identification and implementation of on-line EPI monitoring for these patients.

Although observations from the present study fulfilled our principal objective to determine the clinical feasibility of using an EPID to improve the accuracy of RT administration, this study was not designed to address other important issues. In particular, the protocol did not specify precise tolerance levels for field alignment, as this was at the discretion of the individual radiation oncologist, so we were not able to determine what level of accuracy is attainable with this approach and whether precision is a function of specific parameters (e.g., treatment location, field arrangement, etc.). In addition, we did not compare use of the EPID with portal film procedures to achieve the same objective, and we cannot quantitatively state whether use of the EPID was a time efficient means of accomplishing this task. However, the results of our study lend credence to efforts to address these questions in a series of clinical research efforts to improve the outcomes associated with radiotherapeutic care.

**Conclusion:**

This work demonstrates the feasibility of implementing an EPID-based protocol to improve field alignment in an active radiotherapy practice. The procedure outlined in this report resulted in a reduction in the average SD of setup variation in patients who are difficult to position for radiotherapy to levels that approached those of a control group with no particular setup problems. While pretherapy positioning was not improved for each individual patient from whom this protocol was employed, certain patients had greatly improved positioning as a result of this protocol.

References

1. Killoran JH, Kooy HM, Gladstone DJ, Welte FJ, and Beard CJ. A numerical simulation of organ motion and daily setup uncertainties: implications for radiation therapy*.* Int J Radiat Oncol Biol Phys 1997; 37:213-221.

2. Goitein M, and Busse J. Immobilization error: some theoretical considerations*.* Radiology 1975; 117:407-412.

3. Marks JE, Haus AG, Sutton HG, and Griem ML. Localization error in the radiotherapy of Hodgkin's disease and malignant lymphoma with extended mantle fields*.* Cancer 1974; 34:83-90.

4. Holupka EJ, Humm JL, Tarbell NJ, and Svensson GK. Effect of set-up error on the dose across the junction of matching cranial-spinal fields in the treatment of medulloblastoma*.* Int J Radiat Oncol Biol Phys 1993; 27:345-352.

5. Frazier A, Yan D, Du M, et al. Effects of treatment setup variation on beam's eye view dosimetry for radiation therapy using the multileaf collimator vs. the cerrobend block*.* Int J Radiat Oncol Biol Phys 1995; 33:1247-1256.

6. Niederer J, Hawkins NV, Rider WD, and Till JE. Failure analysis of radical radiation therapy of supraglottic laryngeal carcinoma*.* Int J Radiat Oncol Biol Phys 1977; 2:621-629.

7. Kinzie JJ, Hanks GE, MacLean CJ, and Kramer S. Patterns of care study: Hodgkin's disease relapse rates and adequacy of portals*.* Cancer 1983; 52:2223-2226.

8. Hunt MA, Schultheiss TE, Desobry GE, Hakki M, and Hanks GE. An evaluation of setup uncertainties for patients treated to pelvic sites*.* Int J Radiat Oncol Biol Phys 1995; 32:227-233.

9. Kutcher GJ, Coia L, Gillin M, et al. Comprehensive QA for radiation oncology: report of AAPM Radiation Therapy Committee Task Group 40*.* Med Phys 1994; 21:581-618.

10. Herman MG, Abrams RA, and Mayer RR. Clinical use of on-line portal imaging for daily patient treatment verification*.* Int J Radiat Oncol Biol Phys 1994; 28:1017-1023.

11. Hulshof M, Vanuytsel L, Van den Bogaert W, and van der Schueren E. Localization errors in mantle-field irradiation for Hodgkin's disease*.* Int J Radiat Oncol Biol Phys 1989; 17:679-683.

12. Huddart RA, Nahum A, Neal A, et al. Accuracy of pelvic radiotherapy: prospective analysis of 90 patients in a randomised trial of blocked versus standard radiotherapy. Radiother Oncol 1996; 39:19-29.

13. Rabinowitz I, Broomberg J, Goitein M, McCarthy K, and Leong J. Accuracy of radiation field alignment in clinical practice*.* Int J Radiat Oncol Biol Phys 1985; 11:1857-1867.

14. Rosenthal SA, Galvin JM, Goldwein JW, Smith AR, and Blitzer PH. Improved methods for determination of variability in patient positioning for radiation therapy using simulation and serial portal film measurements [see comments]*.* Int J Radiat Oncol Biol Phys 1992; 23:621-625.

15. Huizenga H, Levendag PC, De Porre PM, and Visser AG. Accuracy in radiation field alignment in head and neck cancer: a prospective study*.* Radiother Oncol 1988; 11:181-187.

16. Michalski JM, Wong JW, Gerber RL, et al. The use of on-line image verification to estimate the variation in radiation therapy dose delivery*.* Int J Radiat Oncol Biol Phys 1993; 27:707-716.

17. Michalski JM, Wong JW, Bosch WR, et al. An evaluation of two methods of anatomical alignment of radiotherapy portal images*.* Int J Radiat Oncol Biol Phys 1993; 27:1199-1206.

18. Marks JE, Haus AG, Sutton HG, and Griem ML. The value of frequent treatment verification films in reducing localization error in the irradiation of complex fields*.* Cancer 1976; 37:2755-61.

19. Valicenti RK, Michalski JM, Bosch WR, et al. Is weekly port filming adequate for verifying patient position in modern radiation therapy? Int J Radiat Oncol Biol Phys 1994; 30:431-438.

20. Shalev S. On-line portal imaging: contributions and limitations in clinical practice*.* Front Radiat Ther Oncol 1996; 29:156-167.

21. Herman MG, Kruse JJ, and Hagness CR. Guide to clinical use of electronic portal imaging*.* J Appl Clin Med Phys 2000; 1:38-57.

22. Luchka K, and Shalev S. Pelvic irradiation of the obese patient: a treatment strategy involving megavoltage simulation and intratreatment setup corrections*.* Med Phys 1996; 23:1897-902.

23. Bel A, Petrascu O, Van de Vondel I, et al. A computerized remote table control for fast on-line patient repositioning: implementation and clinical feasibility*.* Med Phys 2000; 27:354-358.

24. Pisani L, Lockman D, Jaffray D, Yan D, Martinez A, and Wong J. Setup error in radiotherapy: on-line correction using electronic kilovoltage and megavoltage radiographs*.* Int J Radiat Oncol Biol Phys 2000; 47:825-839.

25. Van de Steene J, Van den Heuvel F, Bel A, et al. Electronic portal imaging with on-line correction of setup error in thoracic irradiation: clinical evaluation*.* Int J Radiat Oncol Biol Phys 1998; 40:967-976.

26. Van den Heuvel F, De Neve W, Verellen D, Coghe M, Coen V, and Storme G. Clinical implementation of an objective computer-aided protocol for intervention in intra-treatment correction using electronic portal imaging*.* Radiother Oncol 1995; 35:232-239.

27. Mock U, Dieckmann K, Wolff U, Knocke TH, and Potter R. Portal imaging based definition of the planning target volume during pelvic irradiation for gynecological malignancies*.* Int J Radiat Oncol Biol Phys 1999; 45:227-232.

28. Bel A, Keus R, Vijlbrief RE, and Lebesque JV. Setup deviations in wedged pair irradiation of parotid gland and tonsillar tumors, measured with an electronic portal imaging device*.* Radiother Oncol 1995; 37:153-159.

29. Holmberg O, Huizenga H, Idzes MH, Lebesque JV, Vijlbrief RE, and Mijnheer BJ. In vivo determination of the accuracy of field matching in breast cancer irradiation using an electronic portal imaging device*.* Radiother Oncol 1994; 33:157-166.

30. Creutzberg CL, Althof VG, Huizenga H, Visser AG, and Levendag PC. Quality assurance using portal imaging: the accuracy of patient positioning in irradiation of breast cancer*.* Int J Radiat Oncol Biol Phys 1993; 25:529-539.

31. Meertens H, van Herk M, Bijhold J, and Bartelink H. First clinical experience with a newly developed electronic portal imaging device*.* Int J Radiat Oncol Biol Phys 1990; 18:1173-1181.

32. Ezz A, Munro P, Porter AT, et al. Daily monitoring and correction of radiation field placement using a video-based portal imaging system: a pilot study*.* Int J Radiat Oncol Biol Phys 1992; 22:159-165.

33. Michalski JM, Graham MV, Bosch WR, et al. Prospective clinical evaluation of an electronic portal imaging device*.* Int J Radiat Oncol Biol Phys 1996; 34:943-951.

34. Thompson V, Bidmead M, and Mubata C. Pictorial review: comparison of portal imaging and megavoltage verification films for conformal pelvic irradiation*.* Br J Radiol 1996; 69:1191-1195.

35. Stroom JC, Olofsen-van Acht MJ, Quint S, et al. On-line set-up corrections during radiotherapy of patients with gynecologic tumors*.* Int J Radiat Oncol Biol Phys 2000; 46:499-506.

Figure Legends:

Fig 1. Frequency of Repositioning. Bar graph shows the frequency of protocol patient repositioning based on EPID monitoring. Nine of 14 (64%) of protocol patients required repositioning on greater than 40% of treatment days.

Fig 2. Standard Deviation of Displacement (ML vs. SI). Graph shows the standard deviation (SD) of displacement in the superior-inferior (SI) direction in relation to SD of displacement in the medial-lateral (ML) direction. The distance from the origin to any given point represents the total random variation of setup displacement in the coronal plane for a single patient. Squares = initial SD before correction, triangles = final SD after correction. The reduction in the magnitude of the initial SD to the final SD after use of the protocol is evident.

Fig 3. Standard Deviation of Displacement (AP vs. SI). Graph shows the standard deviation (SD) of displacement in the superior-inferior (SI) direction in relation to SD of displacement in the anterior-posterior (AP) direction. The distance from the origin to any given point represents the total random variation of setup displacement in the sagittal plane for a single patient. Squares = initial SD before correction, triangles = final SD after correction. The reduction in the magnitude of the initial SD to the final SD after use of the protocol is evident.

Fig 4. Maximum Displacement (ML vs. SI). Graph shows the maximum displacement in the superior-inferior (SI) direction in relation to the maximum displacement in the medial-lateral (ML) direction. Squares = initial max displacement before correction, triangles = final max displacement after correction. The reduction in the magnitude of the initial maximum displacement to the final maximum displacement after use of the protocol is evident. Patients with very large maximum displacements initially are seen to benefit most dramatically from the protocol.

Fig 5. Maximum Displacement (AP vs. SI). Graph shows the maximum displacement in the superior-inferior (SI) direction in relation to the maximum displacement in the anterior-posterior (AP) direction. Squares = initial max displacement before correction, triangles = final max displacement after correction. The reduction in the magnitude of the initial maximum displacement to the final maximum displacement after use of the protocol is evident. Patients with very large maximum displacements initially are seen to benefit most dramatically from the protocol.

**Tables:**

Table 1. Characteristics of 14 protocol patients.

|  | Number of Patients |  | % |
| --- | --- | --- | --- |
| Tumor type |  |  |  |
| Cutaneous | 1 |  | 7 |
| Gastrointestinal | 5 |  | 35 |
| Gynecologic | 6 |  | 43 |
| Prostate | 1 |  | 7 |
| Pulmonary | 1 |  | 7 |
| Histological type |  |  |  |
| Adenocarcinoma | 8 |  | 57 |
| Squamous cell carcinoma | 4 |  | 29 |
| Other | 2 |  | 14 |
| AJCC Stage |  |  |  |
| I | 1 |  | 7 |
| II | 5 |  | 35 |
| III | 6 |  | 43 |
| IV | 2 |  | 14 |
| Treatment site |  |  |  |
| Extremity | 1 |  | 7 |
| Pelvis | 12 |  | 86 |
| Thorax | 1 |  | 7 |
| Treatment position |  |  |  |
| Prone | 4 |  | 29 |
| Supine | 10 |  | 71 |
|  |  |  |  |

AJCC: American Joint Commission on Cancer.

Table 2. Magnitude of field displacement in 14 protocol patients.

|  |  | Standard Deviation (mm) | | Maximum (mm) | |  |  |
| --- | --- | --- | --- | --- | --- | --- | --- |
| Patient | Direction | Initial | Final | Initial | Final | Average EPIs/day,  Number (range) | % of days  repositioned |
| 1 | ML | 10.1 | 4.7 | 23.3 | 7.1 |  |  |
|  | SI | 6.4 | 4.2 | 11.9 | 8.0 | 2.3 (1-5) | 75 |
|  | AP | 13.6 | 5.1 | 22.4 | 6.9 |  |  |
| 2 | ML | 1.7 | 2.2 | 2.4 | 3.7 |  |  |
|  | SI | 4.8 | 3.1 | 10.9 | 6.2 | 1.2 (1-3) | 15 |
|  | AP | 5.6 | 3.8 | 9.5 | 9.5 |  |  |
| 3 | ML | 4.6 | 1.9 | 17.0 | 3.9 |  |  |
|  | SI | 3.2 | 3.1 | 7.1 | 7.1 | 1.7 (1-4) | 54 |
|  | AP | 4.4 | 2.6 | 9.8 | 5.1 |  |  |
| 4 | ML | 3.8 | 3.2 | 9.1 | 10.8 | 1.6 (1-6) | 43 |
|  | SI | 4.2 | 4.0 | 12.0 | 9.3 |  |  |
| 5 | SI | 7.4 | 2.7 | 13.3 | 7.9 | 1.6 (1-3) | 33 |
|  | AP | 7.2 | 2.9 | 19.0 | 5.1 |  |  |
| 6 | ML | 3.6 | 2.3 | 7.4 | 4.2 | 1.2 (1-2) | 24 |
|  | SI | 1.1 | 1.0 | 2.7 | 2.5 |  |  |
| 7 | ML | 6.0 | 2.3 | 16.9 | 8.5 | 1.8 (1-3) | 70 |
|  | SI | 1.8 | 1.6 | 4.7 | 4.3 |  |  |
| 8 | ML | 12.5 | 3.2 | 20.9 | 5.9 |  |  |
|  | SI | 4.7 | 5.1 | 11.7 | 10.7 | 2.0 (1-5) | 65 |
|  | AP | 6.6 | 6.9 | 10.8 | 12.2 |  |  |
| 9 | ML | 9.9 | 3.2 | 28.1 | 11.3 | 1.6 (1-4) | 42 |
|  | SI | 3.5 | 3.1 | 8.2 | 8.2 |  |  |
| 10 | ML | 9.1 | 3.0 | 24.8 | 6.8 | 2.1 (1-4) | 86 |
|  | SI | 3.7 | 3.0 | 11.0 | 10.7 |  |  |
| 11 | ML | 10.2 | 2.6 | 22.5 | 7.2 | 3.5 (1-7) | 100 |
|  | SI | 31.0 | 3.4 | 100.8 | 11.2 |  |  |
| 12 | ML | 4.0 | 1.6 | 9.5 | 4.0 | 1.4 (1-2) | 40 |
|  | SI | 5.5 | 0.8 | 13.7 | 2.2 |  |  |
| 13 | SI | 1.6 | 1.5 | 3.4 | 3.4 | 1.2 (1-2) | 21 |
|  | AP | 1.8 | 1.8 | 4.8 | 4.8 |  |  |
| 14 | ML | 7.8 | 6.4 | 17.0 | 15.2 | 1.4 (1-3) | 32 |
|  | SI | 4.2 | 2.8 | 13.2 | 7.1 |  |  |
|  |  |  |  |  |  |  |  |
| Mean | All | 6.4 | 3.1 | 15.6 | 7.2 |  | 50 |
|  |  |  |  |  |  |  |  |

Table 3. Directional displacements for protocol and control patients, with protocol

patients grouped according to frequency of beam re-alignment.

|  |  |  | Standard  Deviation (mm) | |  | Maximum Displacement (mm) | |
| --- | --- | --- | --- | --- | --- | --- | --- |
| Patient Group |  | Direction | Initial | Final | p value | Initial | Final |
| >50% days repositioned |  | ML | 8.8 | 2.9 | 0.002 | 28.1 | 11.3 |
| (n=6) |  | SI | 8.5 | 3.4 | 0.31 | 100.8 | 17.0 |
|  |  | AP | 8.2 | 4.9 | 0.33 | 22.4 | 12.2 |
|  |  |  | (8.5) | (3.5) | 0.0015* |  |  |
|  |  |  |  |  |  |  |  |
| <50% days repositioned |  | ML | 5.1 | 3.2 | 0.11 | 17.0 | 15.2 |
| (n=8) |  | SI | 4.4 | 2.5 | 0.08 | 13.7 | 9.3 |
|  |  | AP | 4.8 | 2.9 | 0.25 | 19.0 | 9.5 |
|  |  |  | (4.6) | (2.7) | 0.002* |  |  |
|  |  |  |  |  |  |  |  |
| No repositioning |  | ML |  | 1.8 |  |  | 7.5 |
| (n=12) |  | SI |  | 1.7 |  |  | 10.8 |
|  |  | AP |  | 2.7 |  |  | 11.9 |
|  |  |  |  | (2.1) |  |  |  |

Mean values are listed in parentheses.

*Overall p value for SD reduction for entire group of patients.

Table 4. Measurement reproducibility in select protocol patients.

|  |  |  | Displacement (mm) | Displacement (mm) |  |  |
| --- | --- | --- | --- | --- | --- | --- |
| Patient | View | Direction | First  Measurement | Second  Measurement |  | Absolute Difference (mm) |
| 2 | AP | ML-initial | 1.7 | 1.7 |  | 0.0 |
|  |  | ML-final | 5.1 | 4.4 |  | 0.6 |
|  |  | SI-initial | 2.2 | 2.0 |  | 0.2 |
|  |  | SI-final | 3.1 | 2.6 |  | 0.5 |
|  | Lateral | AP-initial | 5.6 | 4.9 |  | 0.7 |
|  |  | AP-final | 3.8 | 2.7 |  | 1.1 |
|  |  | SI-initial | 3.8 | 3.1 |  | 0.7 |
|  |  | SI-final | 2.4 | 1.5 |  | 0.9 |
| 5 | Lateral | AP-initial | 7.1 | 5.3 |  | 1.8 |
|  |  | AP-final | 7.0 | 7.4 |  | 0.4 |
|  |  | SI-initial | 3.2 | 6.3 |  | 3.1 |
|  |  | SI-final | 2.8 | 3.0 |  | 0.2 |
| 6 | AP | ML-initial | 3.6 | 3.9 |  | 0.3 |
|  |  | ML-final | 1.1 | 1.0 |  | 0.1 |
|  |  | SI-initial | 2.3 | 2.6 |  | 0.3 |
|  |  | SI-final | 1.0 | 1.0 |  | 0.0 |
| 12 | AP | ML-initial | 4.0 | 3.1 |  | 0.9 |
|  |  | ML-final | 5.5 | 1.7 |  | 3.8 |
|  |  | SI-initial | 1.6 | 3.0 |  | 1.4 |
|  |  | SI-final | 0.9 | 1.3 |  | 0.4 |
| Average difference (mm) | | |  |  |  | 0.9 |
